# Spontaneous Pelvic Rupture as a Result of Renal Colic in a Patient with Klinefelter Syndrome

**DOI:** 10.1155/2013/374973

**Published:** 2013-03-26

**Authors:** Sergey Reva, Yuri Tolkach

**Affiliations:** Urology Clinic, Military Medical Academy, Saint Petersburg 190000, Russia

## Abstract

We present the case of a young man with Klinefelter syndrome, who was admitted to our clinic with renal colic. Shortly after admittance, spontaneous decrease in pain has occurred. Ultrasound and intravenous contrast computed tomography were performed, which showed the evidence of urine extravasation at the level of left renal pelvis and a 4 mm stone in the lower third of the left ureter. The management with a double-J ureteric stent for three weeks was successful. Then, the stent was removed and computed tomography confirmed the absence of urine extravasation. We also analyze the literature related to this case and discuss the main mechanisms of collecting system rupture.

## 1. Introduction

Renal colic is a common urologic emergency affecting 3–5% of the population in the developed countries [1]. The most frequent cause of renal colic is distension of the collecting system from obstructing calculi [2]. Of those patients with ureterolithiasis, depending on stone size and location, approximately 80% will pass the stone spontaneously if managed conservatively [3]. Persistent renal colic leads to complications such as acute infection and renal insufficiency, and intervention may be required [4]. Collecting system rupture is an extremely rare outcome of the increased pressure in the pelvis during renal colic. To date, no explanation of the mechanism and causes of pelvic rupture has been reported in literature the [5]. Theoretically, one possible explanation could be congenital or acquired connective tissue disorders.

## 2. Case Report

A 29-year-old man presented with left renal colic as a complication of nephrolithiasis. The patient was brought in by an ambulance with complaints of acute left flank pain with radiation to the scrotum, nausea, and macroscopic hematuria. This was the first episode of renal colic in his life. At birth, he was diagnosed with a Klinefelter syndrome and was receiving testosterone substitution therapy for the five years before admission. 

Habitus: The patient was tall, slim, narrow shouldered, broad hipped, and with dense and small testes. Slight rebound tenderness to the left lower quadrant suspicious for peritoneal irritation was disclosed on examination; body temperature was 38°C.

Laboratory investigation showed leukocytosis and pyuria. Admission ultrasound disclosed mild hydronephrosis on the left and raised a suspicion for fluid presence in the perirenal fat.

**Figure 1:**
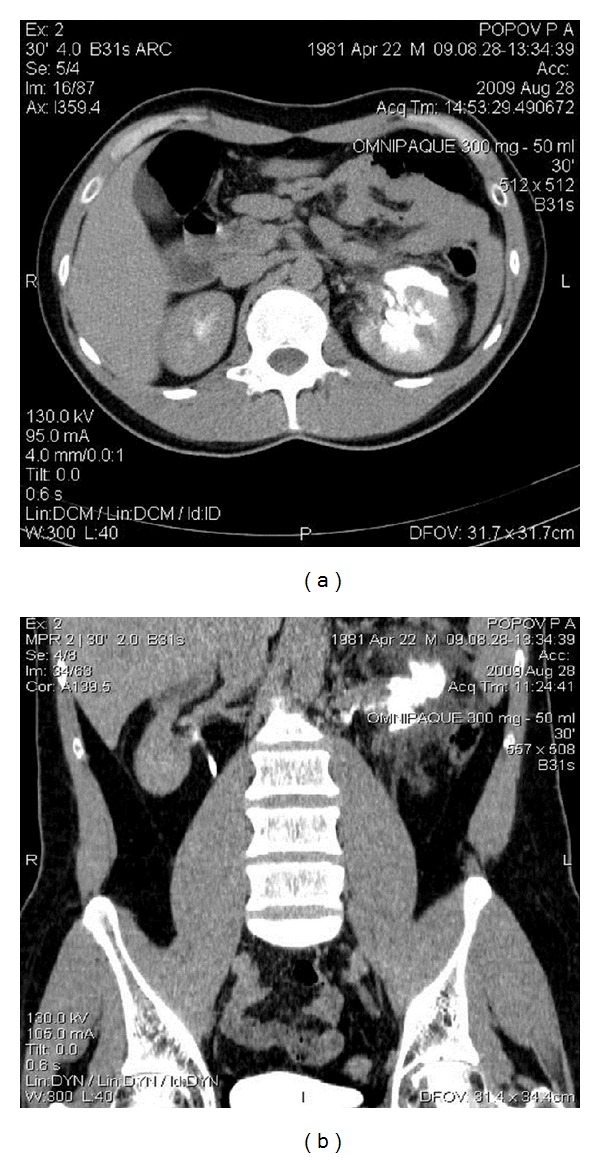
Computed tomography showing extravasation of the contrast medium through the gap in pelvis—spontaneous rupture. Axial (a) and sagittal (b) images.

Almost immediately after admission, before the pain was controlled with intravenous analgesics, there was a spontaneous decrease in pain. Computed tomography (CT) with contrast enhancement was performed 2 hours later and showed distribution of the contrast around the left kidney (Figure 1).

**Figure 2:**
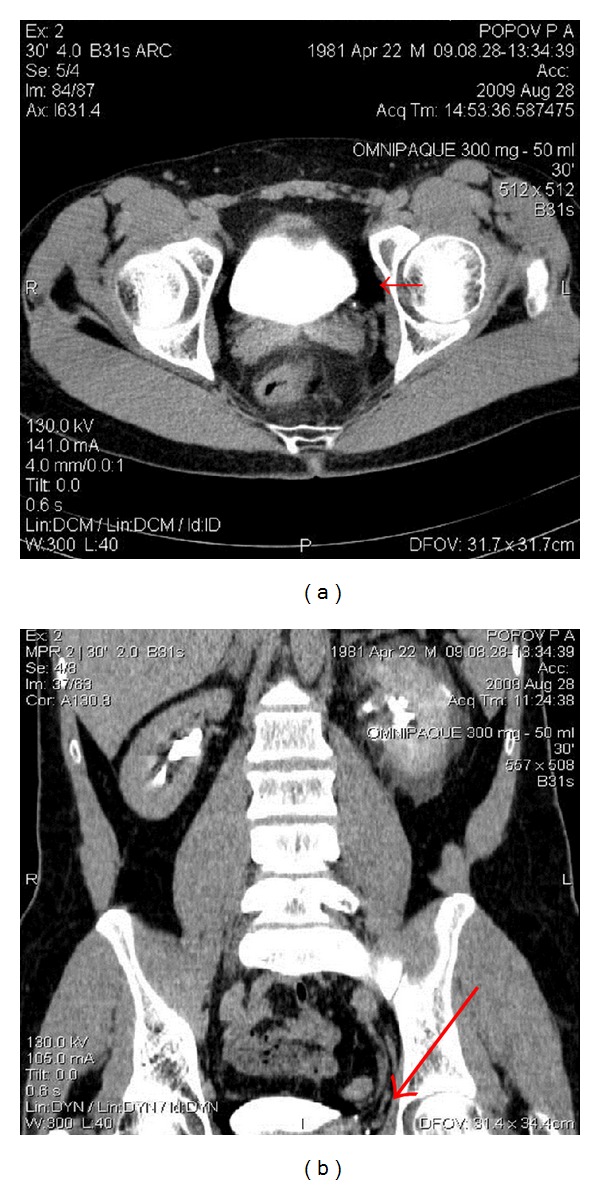
4 mm stone in the lower third of the left ureter (red arrow). Axial (a) and sagittal (b) images.

Also lower third ureter scanning was performed showing a 4-millimeter obstructive stone at the left ureterovesical junction (Figure 2). 

A double-J ureteric stent was placed immediately after tomography without technical problems. On the next day, the patient did not have any discomfort and was discharged from the hospital with the prescriptions for peroral tamsulosin and diclofenac.

Three weeks later, he had no complaints as well. Discharge of stone has not been observed during this period. The red blood cell count, serum creatinine, and urea were normal, and ultrasound of the upper urinary tract was unremarkable.

**Figure 3:**
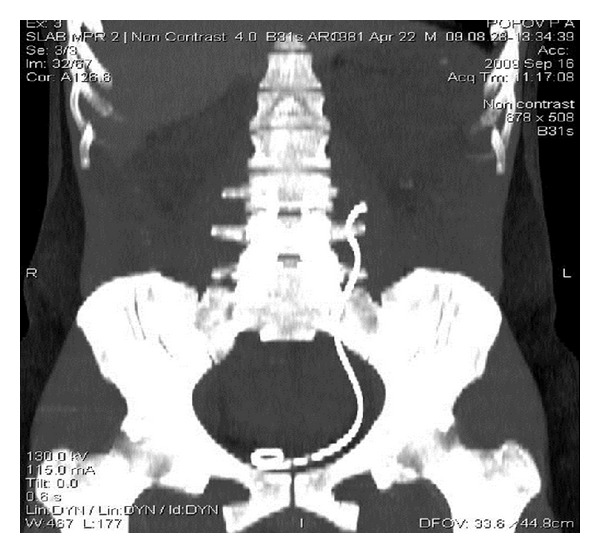
Distal migration of the ureteric stent.

**Figure 4:**
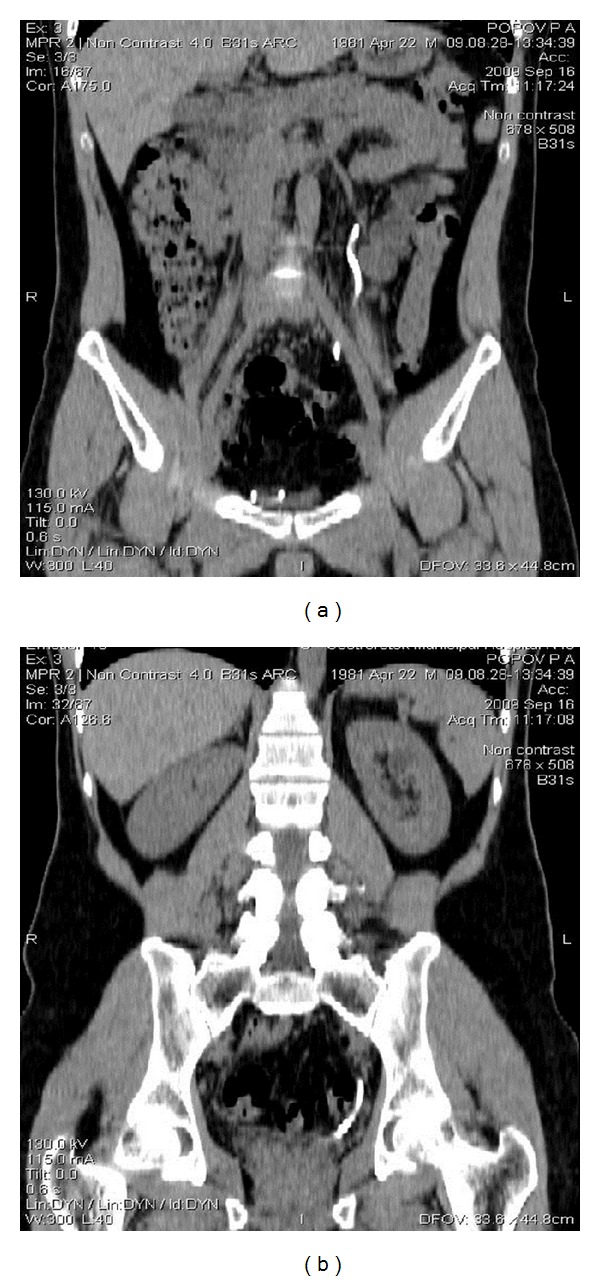
Control CT (3 weeks after stenting and immediately after stent removal)—no extravasation of the contrast medium.

**Figure 5:**
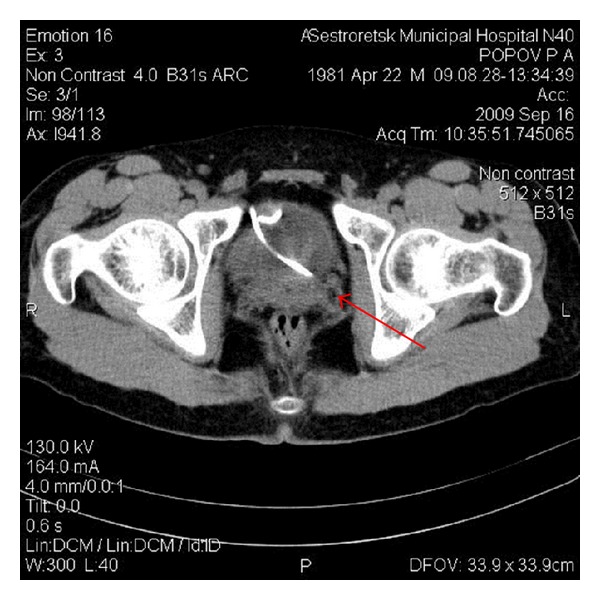
CT showing no stone in the ureter; the area of previous stone location is shown by red arrow.

Control CT showed a partial distal migration of the ureteric stent (Figure 3), which was removed with following CT urography showing no extravasation of the contrast out of the upper urinary tract (Figure 4). On the CT images, there was no evidence of the stone in the urinary tract (Figure 5).

The patient was discharged from the hospital with recommendations (high fluid intake, reduced physical activity, and follow-up examination 3–6 months later).

## 3. Discussion 

There are some data about spontaneous stone passage according to size and localization. The likelihood of expulsion for stones <5 mm is more than 70% [6] and 95% of stones up to 4 mm will pass within 40 days [3]. Moreover, stone expulsion rate depends on the location of the stone; it averages 80% with the location in the lower third regardless of the size [6]. Nevertheless, in our case, it has not happened as alluded above and as a longstanding high-grade obstruction it could contribute to irreversible renal injury. But almost immediately after admission to our hospital, spontaneous decrease of the pain was noted and thereafter a survey revealed a gap in the renal pelvis wall.

Wunderlich in 1856 was the first to describe spontaneous pelvic rupture [7]. Increase of the pressure in the collecting system and tension of the pelvis wall were proposed as the mechanisms of renal colic, and these mechanisms are likely to prevent the damage to the collecting system [8]. Some models revealed that distension-mediated activation of renal pelvis mechanoreceptors results in spinothalamic (pathway) C-fiber excitation. The mean threshold pressure to elicit this response for evoking pain was 30 mm Hg. However, in some cases, pelvic rupture can occur and it could be related to obstruction, trauma, previous urinary tract surgery, or other conditions like hydronephrosis, especially when the renal pelvis is fixed because of fibrosis [5]. Lymphoma and chemotherapy have been described as a cause of spontaneous rupture of the renal pelvis [8]. 

In the present case, nothing of the aforementioned was evident, but the patient had an inherited abnormality Klinefelter syndrome. This is the most common disorder of sex chromosomes in humans, with prevalence of 1 in 500 males, which can lead not only to infertility but to structural alterations in tissues as well [9]. Effects of trophic tissue impairment in Klinefelter syndrome can manifest in different ways—leg ulcers, heart diseases, and rheumatic disease [10]. Hereupon, it is tempting to speculate about the relationship between changes in the wall of the pelvis as a result of Klinefelter syndrome and spontaneous rupture of renal pelvis.

The mechanism of this phenomenon has not been studied, and the case reports in the recent literature are scarce. Probably, this could be associated with decreased muscle layer and fibrotic changes resulting from the compromised tissue oxygen utilization.

As for diagnosis of this complication in this particular case, we have seen the sign of urinary extravasation on ultrasound which warranted CT scan. We consider that in some cases intravenous pyelography could be insufficient for proper diagnosis. Also, we could denote that spontaneous renal colic resolution in the presence of the stone in the ureter could also point at the diagnosis.

Treatment should be individualized in each case. Some studies even describe an open surgery performed for this rare complication [4]. Nevertheless, many authors report on the significant benefits of minimally invasive procedures with double-J ureteral stent placement being a method of choice [5, 8]. 

## 4. Conclusion

Renal pelvis rupture as a result of renal colic is a rare sequel of an otherwise common pathologic process. 

Spontaneous rupture of the renal pelvis is a complication with few characteristic clinical signs. It should be differentiated from other causes of abdominal pain. CT with contrast enhancement is a useful tool in making diagnosis and it could be more sensitive compared to intravenous pyelography. 

The mechanism of spontaneous pelvis rupture has not been studied and probably could be a consequence of the histological alterations. One of such predisposing conditions could be Klinefelter syndrome as it apparently was in our case.

We consider that in most cases this complication should be managed with minimally invasive procedures such as double-J ureteric stent placement. 

## References

[1] Fielding JR, Steele G, Fox LA, Heller H, Loughlin KR (1997). Spiral computerized tomography in the evaluation of acute flank pain: a replacement for excretory urography. *Journal of Urology*.

[2] Miller OF, Kane CJ (1999). Time to stone passage for observed ureteral calculi: a guide for patient education. *Journal of Urology*.

[3] Carter MR, Green BR (2011). Renal calculi: emergency department diagnosis and treatment. *Emergency Medicine Practice*.

[4] Segura JW, Preminger GM, Assimos DG (1997). Ureteral stones clinical guidelines panel summary report on the management of ureteral calculi. *Journal of Urology*.

[5] Diaz ES, Buenrostro FG (2011). Renal pelvis spontaneous rupture secondary to ureteral lithiasis. Case report and bibliographic review. *Archivos Españoles de Urología*.

[6] Bonk JP, Basch RI, Cheris DN (1966). Spontaneous rupture of the renal pelvis. *The American Journal of Roentgenology, Radium Therapy, and Nuclear Medicine*.

[7] Holmlund D (1983). How does a fall in pelvic pressure influence the passage of a ureteral stone?. *Scandinavian Journal of Urology and Nephrology, Supplementum*.

[8] Koktener A, Unal D, Dilmen G, Koc A (2007). Spontaneous rupture of the renal pelvis caused by calculus: a case report. *Journal of Emergency Medicine*.

[9] Giltay JC, Maiburg MC (2010). Klinefelter syndrome: clinical and molecular aspects. *Expert Review of Molecular Diagnostics*.

[10] Shanmugam VK, Tsagaris KC, Attinger CE (2012). Leg ulcers associated with Klinefelter's syndrome: a case report and review of the literature. *International Wound Journal*.

